# INTEGRIN-MEDIATED INTERACTIONS WITH A LAMININ-PRESENTING SUBSTRATE MODULATE BIOSYNTHESIS AND PHENOTYPIC EXPRESSION FOR CELLS OF THE HUMAN NUCLEUS PULPOSUS

**DOI:** 10.22203/eCM.v041a50

**Published:** 2021-06-24

**Authors:** J. Speer, M. Barcellona, L. Jing, B. Liu, M. Lu, M. Kelly, J. Buchowski, L. Zebala, S. Luhmann, M. Gupta, L. Setton

**Affiliations:** 1 Department of Biomedical Engineering, Washington University in St. Louis; St. Louis, MO, USA; 2 Department of Orthopedic Surgery, Washington University School of Medicine; St. Louis, MO, USA

**Keywords:** Intervertebral disc, mechanobiology, biomaterials, signal transduction

## Abstract

With aging and pathology, cells of the nucleus pulposus (NP) de-differentiate towards a fibroblast-like phenotype, a change that contributes to degeneration of the intervertebral disc (IVD). Laminin isoforms are a component of the NP extracellular matrix during development but largely disappear in the adult NP tissue. Exposing human adult NP cells to hydrogels made from PEGylated-laminin-111 (PEGLM) has been shown to regulate NP cell behaviors and promote cells to assume a biosynthetically active state with gene/protein expression and morphology consistent with those observed in juvenile NP cells. However, the mechanism regulating this effect has remained unknown. In the present study, the integrin subunits that promote adult degenerative NP cell interactions with laminin-111 are identified by performing integrin blocking studies along with assays of intracellular signaling and cell phenotype. The findings indicate that integrin α3 is a primary regulator of cell attachment to laminin and is associated with phosphorylation of signaling molecules downstream of integrin engagement (ERK 1/2 and GSK3β). Sustained effects of blocking integrin α3 were also demonstrated including decreased expression of phenotypic markers, reduced biosynthesis, and altered cytoskeletal organization. Furthermore, blocking both integrin α3 and additional integrin subunits elicited changes in cell clustering, but did not alter the phenotype of single cells. These findings reveal that integrin-mediated interactions through integrin α3 are critical in the process by which NP cells sense and alter phenotype in response to culture upon laminin and further suggest that targeting integrin α3 has potential for reversing or slowing degenerative changes to the NP cell.

## Introduction

The IVD is comprised of several major structures, namely the NP, anulus fibrosus, and cartilaginous endplates (Chan *et al.*, 2011; Urban and Roberts, 2003). Together these structures contribute flexibility and motion to the spine, and distribute mechanical forces exerted on the axial skeleton (Fearing *et al.*, 2018; Freemont, 2008; Nachemson, 1981; Neidlinger-Wilke *et al.*, 2014; Roughley, 2004; Setton and Chen, 2006). With aging and degeneration, changes to the IVD are believed to initiate in the NP region including decreased cellularity, hydration, tissue stiffening and a consequent loss of disc height – all of which are hallmarks of a degenerated IVD (Chan *et al.*, 2011; Hoy *et al.*, 2014; Kandel *et al.*, 2008; Weber *et al.*, 2015). Patients presenting with these changes upon radiographic or MR imaging may have impaired physical function, pain upon motion, and associated disability (Hoy *et al.*, 2014; Lim *et al.*, 2005). For these reasons, low-back pain, and degenerative conditions of the IVD represent a major medical and socioeconomic burden (Hoy *et al.*, 2014; Katz, 2006; Ravindra *et al.*, 2018).

The degenerative changes in the NP can be observed across several length scales. In the healthy, juvenile state, the avascular and aneural NP is comprised of notochord-derived NP cells present in a soft matrix (~ 0.5–1 kPa measured in human lumbar discs) (Cloyd *et al*., 2007; Iatridis *et al*., 1996; Iatridis *et al*., 1997) that is highly hydrated (~ 90 % water by wet weight) (Bowles *et al*., 2014; Iatridis *et al*., 1996; Urban and Roberts, 1995). The ECM of the juvenile NP is comprised of collagens (predominantly Type II), proteoglycans (particularly ACAN) and LM, including LM-111, −121, −332, −511 and −521, amongst other non-collagenous proteins (Bowles *et al*., 2014; Chen *et al*., 2009; Hayes *et al*., 2001; Roughley, 2004; Tang *et al*., 2014). In contrast, the degenerative NP is significantly stiffer (10–20 kPa), less hydrated, with a higher ratio of collagen to proteoglycan, and has other alterations to ECM composition (Chen *et al.*, 2009; Cloyd *et al.*, 2007; Gilchrist *et al.*, 2013; Hwang *et al.*, 2014; Iatridis *et al.*, 1997; Urban *et al.*, 2000; Walter *et al.*, 2017). The NP cells themselves are also observed to change with maturation and degeneration – juvenile NP cells are rounded, vacuolated, exist in a clustered morphology, and are biosynthetically active (Bowles *et al.*, 2014; Gilchrist *et al.*, 2013; Risbud *et al.*, 2015; Urban *et al.*, 2000). With maturation and degeneration, cells transition towards a more elongated morphology and express markers of a fibroblast-like phenotype (Bowles *et al.*, 2014; Gilchrist *et al.*, 2013; Risbud *et al.*, 2015; Roughley, 2004; Urban *et al.*, 2000). Together, aging and degeneration result in an NP of lower cell density with a limited capacity for self-repair that can contribute to progressive damage to the IVD, pathology and associated pain and disability (Freemont, 2008; Iatridis *et al.*, 1997; Pfirrmann *et al.*, 2001; Urban *et al.*, 2000).

Both cell-based and biomaterial strategies have been explored as options to treat the degenerated disc towards the goal of slowing or reversing degeneration. Biomaterials have been coupled to many types of ECM proteins in order to regulate phenotype for the entrapped or encapsulated cells giving rise to bioactive materials that may support regeneration for the targeted tissue (Bowles *et al.*, 2014; Choi *et al.*, 2019; Clouet *et al.*, 2019). For the IVD, a large body of work has shown that NP cell phenotype can be modulated through culture on substrates that present LM proteins or LM-mimetic peptides (Barcellona *et al*., 2020; Bridgen *et al*., 2017; Francisco *et al.*, 2013; Francisco *et al*., 2014; Gilchrist *et al*., 2011a; Humphreys *et al*., 2018; Hwang *et al*., 2015). These studies have demonstrated that NP cell behaviors (attachment, morphology, biosynthesis and phenotype) are a function of bulk substrate stiffness and ligand presentation and density (Barcellona *et al*., 2020; Bridgen *et al*., 2017; Francisco *et al*., 2013; Francisco *et al*., 2014; Gilchrist *et al*., 2011a; Humphreys *et al*., 2018; Hwang *et al*., 2015). The mechanisms that mediate NP cell attachment and interaction with LM are not well understood, nor are the pathways that transduce these extracellular cues from the LM-presenting biomaterial to promote gene expression of phenotypic markers consistent with healthy NP cells.

**Fig. 1.**
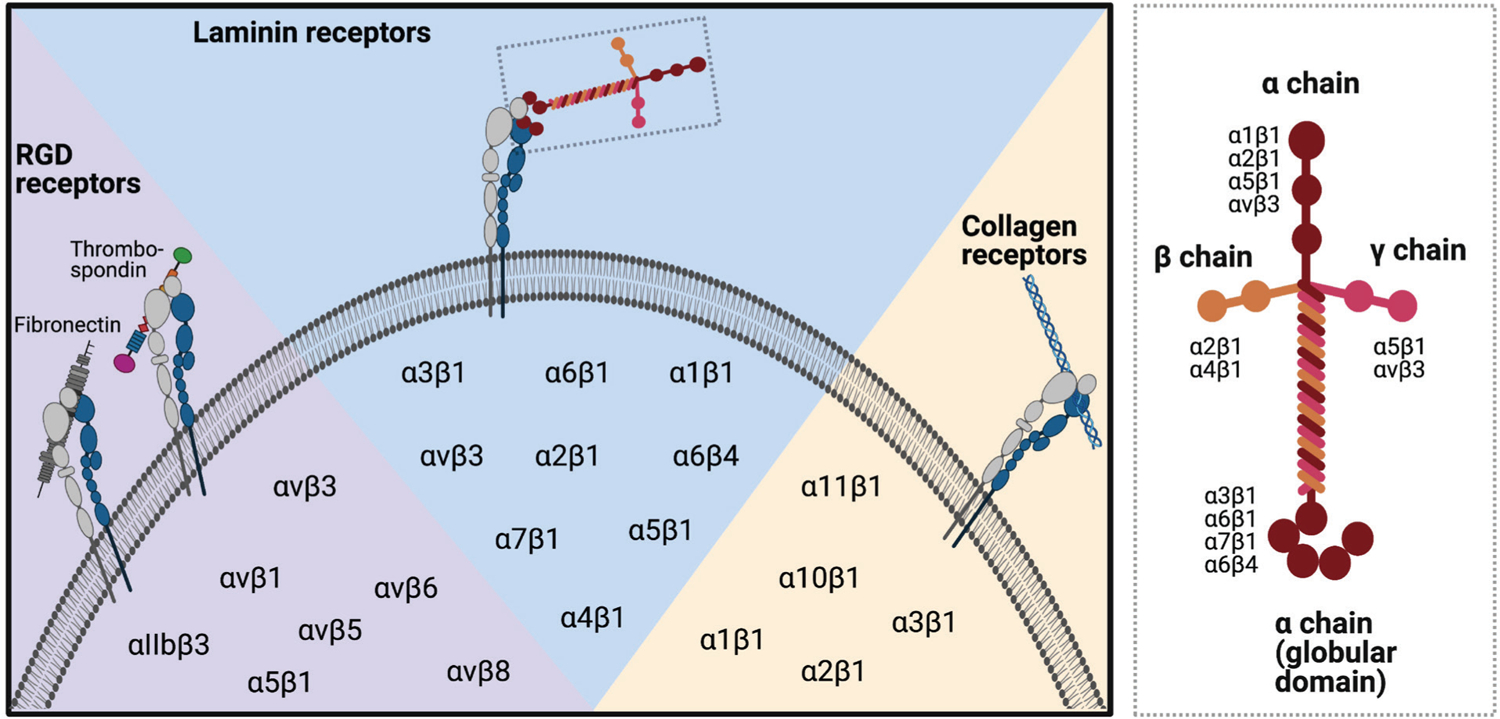
Integrin subunits facilitate cell binding to ECM proteins. Schematic of integrin subunits known to bind ECM proteins (left) and to regions along the laminin protein (right). Created using BioRender.com.

Integrins are well-demonstrated to form mechanosensitive links between proteins in the extracellular domain and the cellular cytoskeleton (Chen *et al*., 2016; Humphries *et al*., 2006; Hynes and Naba, 2012; Winograd-Katz *et al*., 2014). Integrins are heterodimeric proteins made of α and β subunits and specific heterodimers have been shown to bind to particular ECM proteins and peptide sequences (Fig. 1) (Barczyk *et al*., 2010; Belkin and Stepp, 2000; Faull and Ginsberg, 1996; Humphries *et al*., 2006; Hwang *et al*., 2014; Hynes, 1992; Rasmussen and Karsdal, 2016; Takada *et al*., 2007). NP cells have been shown to express several laminin-binding integrin subunits (namely α3, α6, α5, and β1) although the expression of these integrins has been shown to demonstrate variability based on species, age, and pathology (Bridgen *et al.*, 2013; Chen *et al.*, 2009; Gilchrist *et al.*, 2007; Xia and Zhu, 2008). While studies have identified these differences in integrin expression, it remains unclear whether the events of disc degeneration cause altered integrin expression, or whether dysregulation of integrins contributes to disc degeneration. Specifically, Xia and Zhu (2008) have demonstrated that integrins α5 and β1 (but not integrins α1, α2, αv, and β3) were differentially expressed in samples from patients with particular degenerative sub-phenotypes (protrusions and extrusions). Other studies, however, have not found differential expression of integrins α5 and β1 between degenerative and nondegenerative samples (Le Maitre *et al.*, 2009) and additional studies are required to understand the roles and dynamics of integrin expression in the degenerative and regenerative processes.

Data from NP and other cell types (including fibrosarcoma cells, epithelial cells, and keratinocytes) suggest that cells attach to and interact with sequences found within the laminin protein through integrins including α3β1 and α6β1 (Bridgen *et al.*, 2013; Ferletta *et al.*, 2003; Gilchrist *et al.*, 2007; Kim *et al.*, 2005; Mizushima *et al.*, 1997; Nomizu *et al.*, 1995; Nomizu *et al.*, 1997; Nomizu *et al.*, 2000; Velpula *et al.*, 2012; Zhu *et al.*, 2018). Engagement of integrins with ECM proteins triggers activation of signaling pathways including MAPK and ERK 1/2 (Aplin *et al.*, 2001; Ferletta *et al.*, 2003; Francisco *et al.*, 2013; Hood and Cheresh, 2002; Lai *et al.*, 2001; Legate *et al.*, 2009; Saleem *et al.*, 2009; Velpula *et al.*, 2012) and GSK3β/β-catenin (Legate *et al.*, 2009; Ouyang *et al.*, 2017b). These pathways contribute to the regulation of adhesion, gene expression, cell cycle, focal adhesions, and cytoskeletal remodeling, all of which ultimately control cell survival, division, differentiation, and motility (Arboleda *et al.*, 2003; Legate *et al.*, 2009; Ouyang *et al.*, 2017a; Saleem *et al.*, 2009; Winograd-Katz *et al.*, 2014). Altered integrin-regulated mechanotransduction has been observed in degenerative disc cells (Gilbert *et al.*, 2010; Le Maitre *et al.*, 2009; Tsai *et al.*, 2014). Therefore, understanding the integrin expression profiles and integrin-mediated mechanotransduction in adult human degenerative NP cells is needed in order to optimally design materials for the treatment of degenerative disc diseases.

One bioactive material that has been engineered to modulate NP cell behaviors and to support cell delivery to degenerative discs is PEGLM (Fearing *et al.*, 2019; Francisco *et al.*, 2013; Francisco *et al.*, 2014). Prior work has shown that cells interacting with PEGLM exhibit higher biosynthesis of sulfated glycosaminoglycans and markers of the juvenile NP phenotype, suggesting that it is the laminin ligand presentation that is responsible for the effect (Fearing *et al.*, 2019; Francisco *et al.*, 2013; Francisco *et al.*, 2014); however, the mechanism by which NP cells interact with laminin in this biomaterial and the regulatory effects upon phenotype and synthesis remain unknown. The present study aimed to test the hypothesis that NP cells interact with the laminins in PEGLM through integrin engagement and subsequent signal transduction through the ERK and GSK3β pathways. Furthermore, the specific integrin subunits critical for driving the transcription of genes and translation of proteins in degenerative human NP cells that support cell morphology, biosynthesis, and markers of the juvenile NP cell phenotype when interacting with this biomaterial were identified.

## Materials and Methods

### Primary human NP cell culture

NP tissue was obtained from to-be-discarded surgical waste tissues (exempt from IRB review, Washington University Institutional Review Board) of patients (ages 16–75, male and female) receiving surgical treatment for degenerative conditions of the IVD; only demographic information was collected on each patient (age, race, and sex). NP cells were enzymatically isolated from tissues, as previously described (Bridgen *et al.*, 2017; Fearing *et al.*, 2019). In brief, the NP tissue was digested for 2–4 h at 37 °C [0.4 % collagenase type II (Worthington Biochemical; Lakewood, NJ, USA), 0.2 % pronase (Roche; Basel, Switzerland), and 5 % FBS; 23 mL/g tissue]. Isolated NP cells were passed through a 70 μm filter and then expanded in monolayer culture using Ham’s F12 media (Life Technologies; Carlsbad, CA, USA) supplemented with 1 % penicillin/streptomycin and 10 % FBS under 5 % CO_2_ and atmospheric O_2_ at 37 °C. Cells were not used for experimentation past passage 4, and all experiments were conducted using at least 3 human subjects (biological replicates); assay-specific samples sizes are detailed below.

### PEGLM synthesis and coating of tissue culture surfaces

PEGLM was prepared as previously described (Fearing *et al.*, 2019; Francisco *et al.*, 2013). Laminin-111 (LM-111, Trevigen; Gaithersburg, MD, USA) was reacted with acrylate-PEG-hydroxysuccinimide (Ac-PEG-NHS, 10 kDa, Creative PEGWorks; Winston-Salem, NC, USA) in order to form PEGLM. This solution was then dialyzed against PBS in order to remove unreacted Ac-PEG-NHS groups and PEGLM concentration was determined at 280 nm absorbance. Stiff (> 1 GPa) tissue culture surfaces (*i.e*., polystyrene well plates or glass chamber slides; Nunc Lab-Tek Chamber Slide Systems, Thermo Fisher Scientific; Waltham, MA, USA) were coated with the PEGLM solution (diluted with sterile PBS to a working concentration of 22.5 μg/mL). The PEGLM was allowed to adsorb to the culture surface overnight at 4 °C. The following day, the solutions were removed, and surfaces were rinsed with sterile PBS prior to inception of the experiment.

### Functional inhibition of integrin subunits

#### Single integrin blocking

Following detachment from culture plastic, NP cells were treated with blocking antibodies in order to reduce integrin functionality. For antibody-based inhibition, NP cells were incubated with azide-free antibodies (20 μg/mL) targeting integrin α3, α4, α5, αv, α6, or β1 subunits or the respective IgG control antibody for 30 min on a rocker at 37 °C (Table 1) in a manner consistent with previously published integrin-blocking studies in NP and other cell types (Bridgen *et al*., 2013; Ferletta *et al*., 2003; Gilchrist *et al*., 2007). Following this incubation period, the cell solutions containing the antibody were then seeded directly onto the culture substrates.

#### Double integrin blocking

In order to determine if compensatory cell interactions with LM could occur through competing integrin subunits, double integrin-blocking experiments were performed. Single integrin blocking was conducted as before by incubating the assay-appropriate number of cells with 20 μg/mL of the respective antibody for 30 min before seeding the cells (in the antibody-containing media) on PEGLM-coated substrates. After 24 h of culture, additional antibody (20 μg/mL, α3 or additional integrin subunits α5 or α6) was added into the well without removing the prior antibody or media. 24 h after adding the second antibody, cells were fixed in 4 % PFA and prepared for subsequent analysis.

### Cell attachment

As described previously, wells of a 96-well ½-area plate were coated with PEGLM overnight. Serum-starved NP cells were detached from culture plastic using trypsin that was then neutralized with trypsin soybean inhibitor. The cells were then resuspended in serum-free F-12 medium and incubated with integrin-blocking or isotype-matched control antibodies (20 μg/mL) for 30 min before being seeded into wells of the 96-well ½-area plate, at a density of 8,000 cells per well, in a manner consistent with previous studies (Barcellona *et al*., 2020; Bridgen *et al*., 2013; Ding *et al*., 2014; Hiyama *et al*., 2010). After 2 h, cell attachment was quantified through the use of the CellTiter-Glo (Promega; Madison, WI, USA) plate-reader assay (EnSpire Multimode plate reader, PerkinElmer; Waltham, MA, USA) following manufacturers’ and previously established protocols (Barcellona *et al*., 2020; Bridgen *et al*., 2013; Ding *et al*., 2014). Relative cell attachment was defined as 100 × (integrin-blocked sample/IgG-treated sample). Paired one-tailed *t*-tests were used to test the hypothesis that integrin-blocked samples would exhibit reduced cell attachment compared to cells treated with the isotype control. Relative cell attachment values below 50 % of the IgG were considered to be meaningfully reduced based on prior findings (Ferletta *et al*., 2003; Gilchrist *et al*., 2007).

### Protein phosphorylation

Phosphorylation of the proteins ERK 1/2 (Thr202/Tyr204), and GSK3β (Ser9) were quantified using AlphaLISA SureFire Ultra Assay Kits (high volume, PerkinElmer). During protocol development, the optimal time to observe maximal protein phosphorylation was determined by first comparing phosphorylation of proteins in primary NP cells cultured on PEGLM to those on BSA-coated controls or in suspension. Maximal protein phosphorylation in degenerative adult NP cells was found to occur at 15 min for ERK 1/2 and 120 min for GSK3β, time points that corroborate prior findings in the literature (Aplin *et al.*, 2001; Blaustein *et al.*, 2013; Ferletta *et al.*, 2003; Francisco *et al.*, 2013; Grzesiak *et al.*, 2005; Tsai *et al.*, 2007); thus, these respective times were incorporated into the protocols for all future experiments.

NP cells were detached from culture plastic using trypsin that was neutralized with trypsin-soybean inhibitor and resuspended in serum-free F-12 medium. Following incubation with integrin-blocking antibodies, 10,000 NP cells were seeded into wells of a 96-well ½-area plate (coated with PEGLM) and allowed to incubate for 15 or 120 min, as appropriate. After the incubation period the AlphaLISA assay was performed per manufacturer’s protocol. Briefly, medium was removed, and lysis buffer was added to each well. The AlphaLISA reaction buffers (donor and acceptor mixes) were then added to the lysate before wells were read using the plate reader (EnSpire Multimode) using the AlphaScreen protocol (excitation at 680 nm, emission at 615 nm). Relative phosphorylation was defined as 100 × (integrin-blocked sample/IgG-treated sample). Paired one-tailed *t*-tests were used to test the hypothesis that integrin-blocked samples would exhibit reduced protein phosphorylation compared to cells treated with the isotype control. Additionally, phosphorylation below a value equivalent to 50 % of the IgG was considered to be meaningfully reduced, based on prior findings (Ferletta *et al*., 2003; Saleem *et al*., 2009).

### Immunocytochemistry and image analysis

NP cells were seeded at a density of 8,000–10,000 cells per chamber well and cultured for 24 h-4 d; immunolabeling for baseline integrin expression (no integrin blocking) was performed after 24 h of culture on untreated polystyrene *(i.e.*, prior to exposure to PEGLM); additional immunolabeling following integrin-blocking and culture on PEGLM was performed at 4 d of culture. After duration of culture, samples were fixed in 4 % PFA for 10 min, permeabilized using 0.2 % Triton X-100 in PBS^+/+^ for 10 min and incubated for 30 min in blocking buffer (3.75 % BSA and 5 % goat serum, Thermo Fisher Scientific), all at room temperature. Samples were then immunolabeled with antibodies to detect protein expression for markers of the NP cell phenotype (Risbud *et al.*, 2015) and integrin subunits (Table 1 and Table 2) or the respective isotype-matched controls (IgG), overnight at 4 °C. Additional samples were stained with rabbit-anti-Paxillin (1 : 100, Abcam) to quantify focal adhesion formation and/or Alexa-conjugated phalloidin (1 : 200) to stain F-actin in the cell’s cytoskeleton. AlexaFluor (Invitrogen; Carlsbad, CA, USA) secondary antibodies were applied at a dilution of 1 : 200 and nuclei were counterstained with DAPI (2 μg/mL, Sigma-Aldrich).

Samples were imaged using confocal microscopy (TCS- SPE with DM6 RGBV confocal microscope; Leica DFC7000 T camera; using Leica LAS X core software; Leica Microsystems) and a 20 × objective. Images were analyzed using Fiji software (Schindelin *et al.*, 2012) to quantify morphology metrics (*e.g.,* circularity, spread area), mean fluorescence intensity of cells or cell clusters, analysis of actin fibers, and characterization of paxillin-rich focal adhesions according to established protocols (Fearing *et al.*, 2019; Horzum *et al.*, 2014; Püspöki *et al.*, 2016; Sage *et al.*, 2005; Schindelin *et al.*, 2012). Percent of positive cells were defined as cells with MFI greater than that of the control (IgG) antibody used for immunostaining. Relative protein expression was calculated as 100 × (integrin-blocked/average value of the respective control). Upregulated and downregulated protein expression following integrin blocking was defined as cells/cell clusters with MFIs showing a 50 % increase or decrease in expression compared to the average value for cells in the control group, respectively. For quantification of integrin expression, a minimum of 180 cells were evaluated as obtained from 4–8 biological replicates per integrin subunit. Morphology (% clustered, cell spread area, and circularity), cytoskeletal organization, and phenotypic protein expression was characterized by assessing a minimum of 25 cells per treatment group (IgG-treated or integrin-blocked) from 3 biological replicates. Lastly, focal adhesion formation was analyzed in at least 30 cells per treatment group as obtained from 3 biological replicates. For single integrin blocking studies, one-tailed unpaired *t*-tests were used to test for differences in cell spread area, circularity, protein expression, actin alignment, or focal adhesion parameters between integrin-blocked samples and the isotype control. Specifically, it was hypothesized that integrin blocking would promote a shift away from the healthy NP cell phenotype previously observed in culture on laminin-substrates (Barcellona *et al*., 2020; Francisco *et al*., 2014; Gilchrist *et al*., 2011a) as seen by increased cell spread area, decreased circularity, and decreased protein expression of phenotypic markers. Furthermore, as integrin blocking alters interactions with the ECM, it was hypothesized that compared to IgG-treated cells, cells subjected to integrin blocking would demonstrate reduced actin alignment and fewer elongated focal adhesions (Besser and Safran, 2006; Kim and Wirtz, 2013). Two-tailed *t*-tests were used to test for differences in integrin expression following integrin α3 blocking and a one-way ANOVA with Dunnett’s *post-hoc* test was performed to test for differences between double integrin-blocked samples compared to samples blocked with integrin α3 alone. Additionally, Fisher Exact Tests with a Freeman-Halton extension were used to test for differences in the distribution of cell morphologies between the treated and control groups. This statistical test was run on a 2 × 3 contingency table based on the number of cells adherent as single cells, small clusters, or large clusters in experimental groups (*i.e.,* IgG-treated compared to integrin α3 or integrin α3 compared to integrin α3+ α5 or integrin α3+ α6).

### Quantification of gene expression using RT-qPCR

NP cells were detached from culture plastic using trypsin, neutralized with F-12 media, subjected to integrin blocking, and seeded into PEGLM-coated chamber slide wells at a density of 300,000 cells per well. Following 4 d of culture on PEGLM, NP cells were lysed using RLT buffer (Qiagen; Hilden, Germany) with 1 % β-mercaptoethanol. RNA extraction was conducted using an RNeasy mini kit with DNase I digestion (Qiagen; Hilden, Germany) according to the manufacturer’s protocol. The RNA was read for concentration and quality (260 nm/280 nm, NanoDrop One Spectrophotometer, Thermo Scientific; Waltham, MA, USA) and then reverse transcribed into cDNA (iScript cDNA synthesis kit, Biorad; Hercules, CA, USA); qPCR was used to determine expression levels for *ACAN*, *COL2A1*, *CDH2*, *GLUT1*, and integrin subunits (*ITGB1*, *ITGB4*, *ITGA6*, *ITGA10*, and *ITGA11*) compared to housekeeping genes (*GAPDH* and *18S*); a reference sample (cells treated with the isotype control antibody) was included with each RT-qPCR reaction. mRNA was isolated from cells of 6–8 biological replicates per target. Values for fold-difference between each sample and reference sample were calculated as 2^−ΔΔCt^. Paired one-tailed *t*-tests (of ΔCt values) were used to test the hypothesis that integrin blocking would reduce expression of phenotypic markers of NP cells (*ACAN*, *COL2A1*, *CDH2*, and *GLUT1*) compared to the IgG control group. The impact of integrin blocking on the expression of integrin subunits was also evaluated using a two-tailed paired *t*-test comparing integrin-blocked samples to the control group.

### Analysis of biosynthesis and visualization of nascent proteins

The production of sGAG was quantified for NP cells cultured on PEGLM that had been subjected to integrin blocking or incubation with isotype control antibodies using the DMMB spectrophotometric assay (Farndale *et al*., 1982; Gilchrist *et al*., 2011a). The media were collected from wells of NP cells (30,000 cells per well) that had been in culture for 4 d before adding papain solution (125 μg/mL in PBS with 5 mmol/L EDTA and 5 mmol/L L-cysteine) for 2 h at 50 °C to digest the sample. The amount of sGAG in the medium overlay and the papain-digested samples was quantified by comparing absorbance readings (525 nm) against a chondroitin sulfate standard (Sigma-Aldrich) and a medium-only control for correction against colorimetric interference. Total sGAG was determined by adding the results of the medium overlay and the digested cell sample, and then normalized to DNA content as quantified by the Quant-iT PicoGreen dsDNA kit (Invitrogen), following manufacturer’s protocol. A total of 6 samples were evaluated, as obtained from 3 biological replicates. Relative biosynthesis or DNA content was calculated as 100 × (integrin α3 blocked/IgG-treated). Paired one-tailed *t*-tests were used to test the hypothesis that integrin blocking would reduce sGAG production, but not DNA content, compared the respective control.

Production of newly synthesized proteins was further assessed through use of a FUNCAT approach (Dunham *et al.*, 2020; McLeod and Mauck, 2016; tom Dieck *et al.*, 2015), based on measuring an amino acid analog capable of being incorporated into newly synthesized proteins. A fluorescently tagged molecule (Dibenzocyclooctyne, DBCO; Click Chemistry Tools; Scottsdale, AZ, USA) was used to tag the amino acid analog of methionine, allowing for the visualization of proteins synthesized since the analog was present in the medium. Briefly, 2 medium solutions were prepared for use in cell culture – DMEM (without L-glutamine, sodium pyruvate, HEPES, L-methionine, L-cysteine) was supplemented with 10 % FBS, sodium pyruvate (Thermo Fisher Scientific), ascorbic acid (Sigma-Aldrich), glutamax (1 : 100, Thermo Fisher Scientific), 1 % penicillin/streptomycin, L-cysteine (Sigma-Aldrich), and either L-methionine (control media, Sigma-Aldrich) or AHA (L-azidohomoalanine, an analog for L-methionine used for the labeling media, Click Chemistry Tools). Cells were removed from tissue culture flasks and resuspended in either of these two media, subjected to integrin blocking, and seeded into PEGLM coated wells as before (30,000 cells per well). After 4 d of culture on PEGLM, the culture medium was removed and all samples were incubated with DBCO-488 (5 mmol/L DBCO-488 diluted 1 : 165 in PBS with 1 % BSA) for 40 min under cell culture conditions (37 °C, 5 % CO_2_, atmospheric O_2_). Afterwards, the samples were washed, fixed with 4 % PFA (10 min, room temperature), and stained with AlexaFluor phalloidin-633 and DAPI (2 μg/mL) to visualize cytoskeleton and nuclei. The samples were imaged, as previously described, for immunostaining (SPE DM6 Leica confocal microscope). The production of new intracellular proteins was assessed by measuring the mean fluorescence intensity for the region within the cell body (as determined by the localization of phalloidin stain) while extracellular protein synthesis was quantified by examining the fluorescence located outside of the cell body. A minimum of 18 cells per treatment group were evaluated as obtained from 3 biological replicates. Unpaired one-tailed *t*-tests were used to test for reduced nascent protein production (using MFI) both within and outside the cell in the integrin-blocked samples compared to the IgG control.

## Results

### Degenerative human NP cells express integrin α3, α4, α5, αv, α6, and β1 subunits

**Fig. 2.**
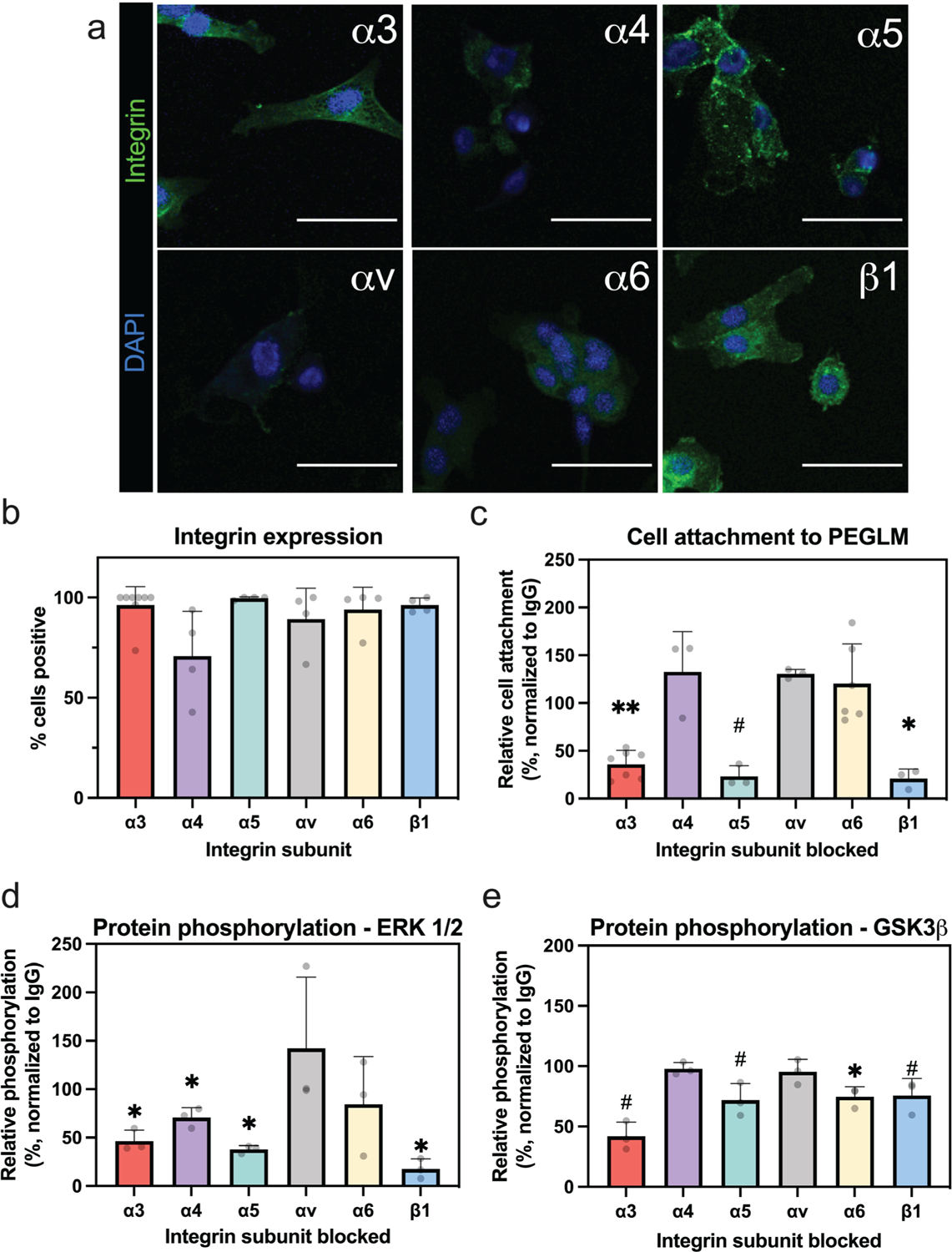
Adult degenerative NP cells express integrin subunits that facilitate interactions with PEGLM. (**a**) Expression of integrin subunits as shown in representative images (scale bar = 50 μm) and (**b**) quantification of the percentage of cells positive for given integrin subunits in NP cells cultured on untreated polystyrene. (**c**) Relative NP cell attachment to PEGLM-coated polystyrene following integrin-blocking compared to IgG controls, relative attachment = 100 × (integrin-blocked/IgG-treated). Relative protein phosphorylation of (**d**) ERK 1/2 and (**e**) GSK3β in cells treated with function-blocking antibodies compared to cells treated with IgG control when seeded on PEGLM-coated polystyrene, relative phosphorylation = 100 × (integrin-blocked/IgG-treated). For all data presented: error bars = standard deviation; each dot = data from 1 biological replicate; ** *p* < 0.01, * *p* < 0.05, # *p* < 0.09 based on results of paired one-tailed *t-*tests (integrin-blocked compared to IgG-treated).

NP cells isolated from degenerative human IVD tissues were observed to express integrin α3, α4, α5, αv, α6, and β1 subunits with greater than 70 % of cells expressing each protein respectively following culture for 24 h on untreated polystyrene (Fig. 2a,b). These findings corroborate results from prior studies which have quantified protein expression of integrin subunits in human NP cells using antibody-based staining and/or flow cytometry (Bridgen *et al*., 2013; Chen *et al*., 2009; Le Maitre *et al.*, 2009). While inter-patient variability was observed in integrin expression, the data did not demonstrate an association with patient age, nor were differences observed as a function of the patient’s sex (data not shown).

### Integrin α3β1 plays a consistent role in regulating NP cell interactions with LM substrates at early time points

NP cell attachment to PEGLM was reduced to less than 50 % of the attachment observed in the IgG-treated group when integrins α3 or β1 were blocked using targeted antibodies (*p* = 0.015 and *p* = 0.045, respectively, Fig. 2c). Blocking integrin α5 reduced cell attachment to levels below 50 % of that seen in the IgG group, though the comparison to this control trended towards significance (*p* = 0.059, Fig. 2c). In contrast, cell attachment was not reduced when integrins α4, αv, or α6 were blocked (Fig. 2c; *p* = 0.19, *p* = 0.12 and *p* = 0.22, respectively).

The binding of integrins to ECM proteins is known to initiate intracellular signaling cascades including the phosphorylation of ERK 1/2 and GSK3β that drive cell survival, proliferation, gene expression, motility, and other cellular functions. For cells seeded onto PEGLM, inhibiting integrins α3, α4, α5, or β1 with function blocking antibodies significantly reduced ERK 1/2 protein phosphorylation (Fig. 2d, *p* = 0.028, *p* = 0.025, *p* = 0.033, and *p* = 0.016, respectively); however, only blocking integrins α3, α5, or β1 reduced phosphorylation below the 50 % threshold. In contrast, blocking integrins αv and α6 did not significantly reduce ERK 1/2 phosphorylation (*p* = 0.20 and *p* = 0.22, respectively, Fig. 2d). GSK3β phosphorylation was reduced (significantly or at levels that trended to significance) in cells where integrins α3, α5, α6, and β1 were inhibited (*p* = 0.051, *p* = 0.0651, *p* = 0.041, and *p* = 0.086, Fig. 2e) but was not reduced by blocking α4 or αv (*p* = 0.31 and *p* = 0.29, Fig. 2e). Only inhibiting integrin α3 reduced GSK3β to levels below the 50 % threshold. Taking the results of cell attachment and protein phosphorylation together, it can be seen that multiple integrin subunits can contribute to early NP cell interactions with PEGLM. However, only blocking integrin α3 resulted in reductions below the 50 % threshold for cell attachment and phosphorylation of both proteins and thus the role of this integrin was further explored in subsequent experimentation.

### Treatment of NP cells with antibodies to block integrin α3 alters cell morphology, cytoskeletal organization, and focal adhesions

**Fig. 3.**
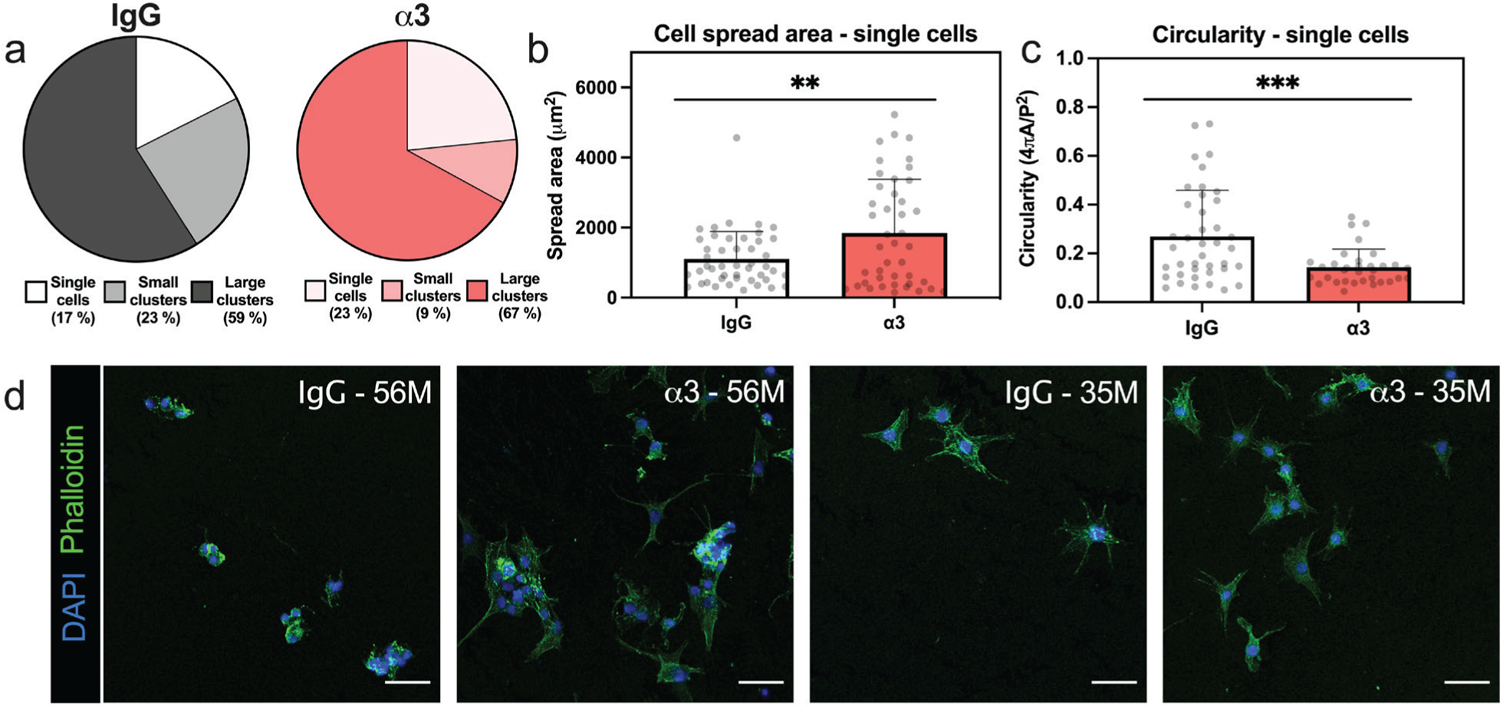
Antibody blocking of integrin α3 alters cell morphology and shape. Characterization of cells treated with an α3-inhibiting or IgG control antibody: (**a**) % of cells attaching to PEGLM as single cells, small clusters (2–3 cells), or large clusters (4+ cells) (**b**) cell spread area (μm^2^) of single cells and (**c**) circularity of single cells. (**d**) Representative images of cell morphology in the IgG or integrin-blocked groups for cells from 2 biological replicates (56M and 35M). For all data presented: error bars = standard deviation; each dot = 1 cell as obtained from *n* = 3 biological replicates; *** *p* < 0.001, ** *p* < 0.01, based on results of unpaired one-tailed *t-*tests comparing integrin α3-blocked to IgG control.

Adult degenerative NP cells treated with a function blocking antibody targeting integrin α3, demonstrated differences in distributions of cellular morphology compared to those treated with an isotype-control antibody (*p* = 0.023, Fig. 3a,d). In comparison to the control group, cells treated with the α3 antibody were observed to form fewer small clusters (2–3 cells) but attached to PEGLM more frequently as either single cells or large clusters (4+ cells). For cells that attached to the PEGLM as single cells, inhibition of integrin α3 resulted in increased cell spread areas and decreased circularity (*p* = 0.0024, Fig. 3b; *p* = 0.0003, Fig. 3c).

**Fig. 4.**
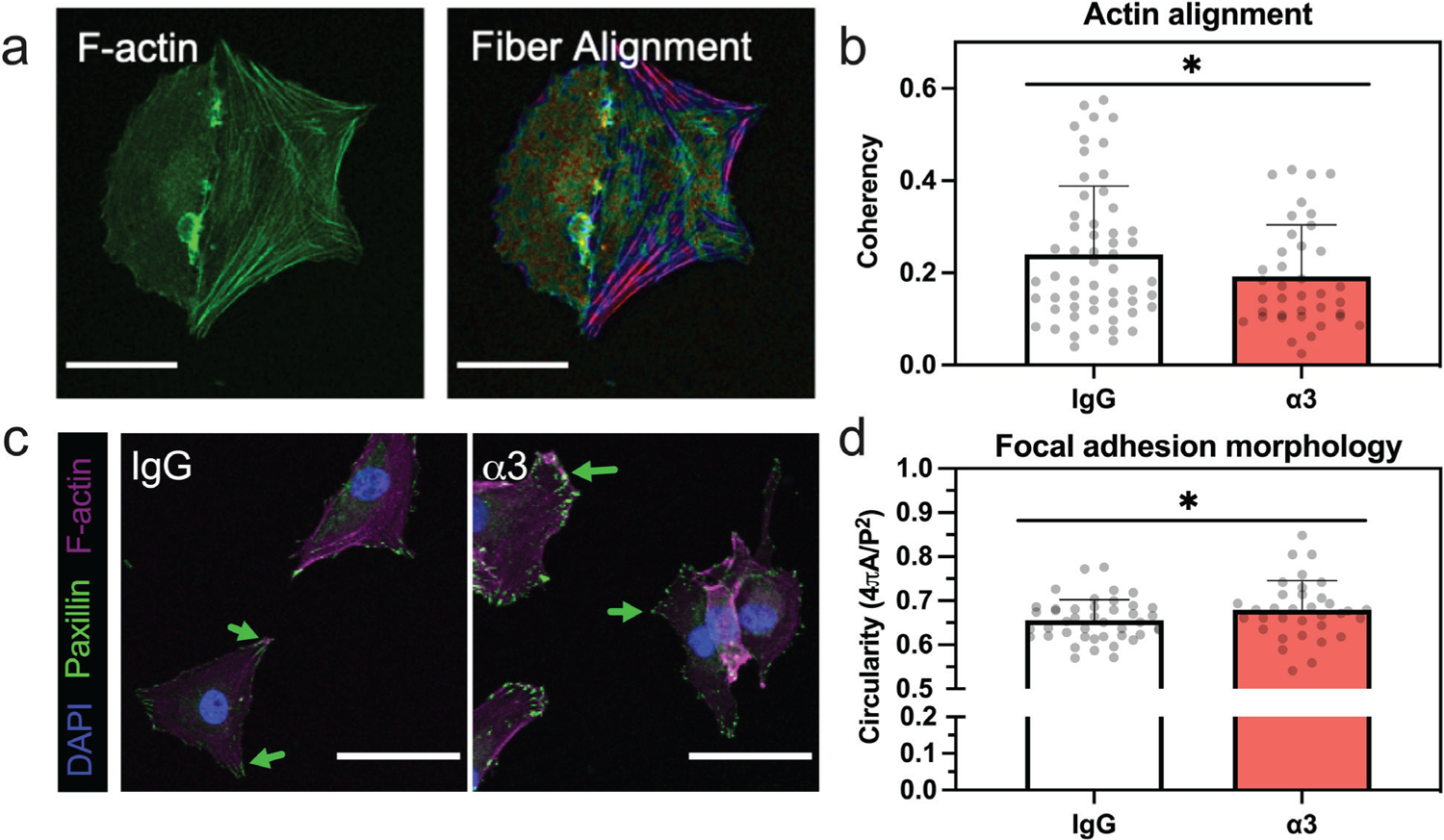
Inhibition of integrin α3 alters actin alignment and focal adhesion morphology. (**a**) Representative images of phalloidin-stained F-actin (green) and processing using OrientationJ to calculate fiber alignment (color coding shows heat map of actin alignment; scale bar = 50 μm). (**b**) Analysis of extent of fiber alignment (coherency) for single cells in the IgG-treated or α3-blocked conditions where a value of 0 corresponds to unaligned fibers and a value of 1 represents perfectly aligned fibers. (**c**) Representative images of paxillin staining with nuclei and F-actin counterstained; scale bar = 50 μm, arrows indicate elongated FA formation in the IgG condition and round FA in the α3-blocked cells. (**d**) Circularity of focal adhesions in cells treated with IgG or α3-targeting antibodies. For all data presented: error bars = standard deviation; each dot = 1 cell as obtained from *n* = 3 biological replicates; * *p* < 0.05 based on results of unpaired one-tailed *t-*tests (comparing IgG-treated to integrin-blocked).

In addition to changes in shape, actin fiber alignment (coherency) was reduced in single cells subsequent to the impairment of integrin α3 (*p* = 0.047, Fig. 4a,b). Paxillin-positive FA were seen in both the integrin inhibited and control cells (Fig. 4c). Although an increase in FA circularity was observed in the integrin-blocked group (*p* = 0.033, Fig. 4d). These observed differences in cytoskeletal structure, while statistically significant, were modest. However, recent studies on NP cells have demonstrated that altered actin alignment, even when relatively slight, can result in significant changes to NP cell phenotype (Barcellona *et al*., 2020; Fearing *et al*., 2020). For example, inhibition of YAP signaling, and a subsequent promotion of cell rounding, reduced actin coherency ~ 60 % and resulted in the differential regulation of over 9,000 genes in degenerative human NP cells (Fearing *et al*., 2020).

### Inhibition of NP cells with antibodies to block integrin α3 alters biosynthesis

**Fig. 5.**
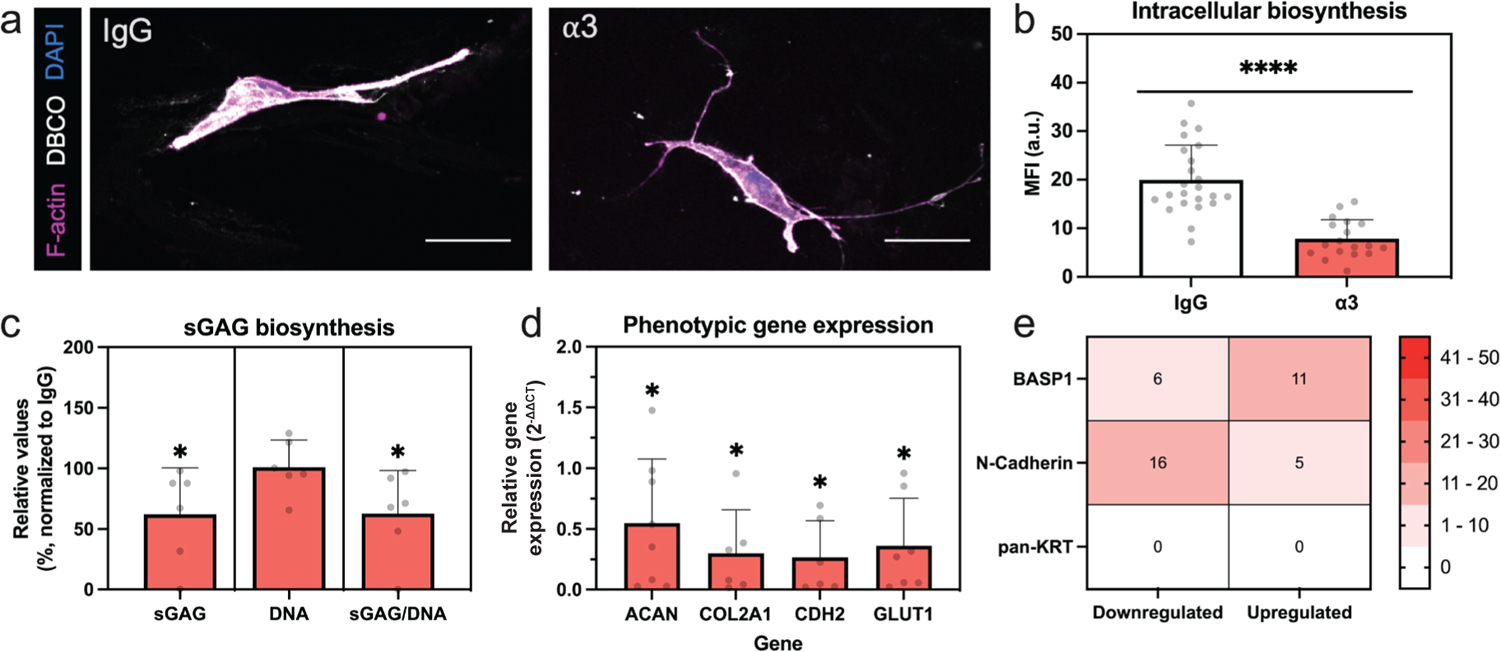
Blocking integrin α3 reduces biosynthesis and expression of phenotypic markers. (**a**) Representative images using FUNCAT staining (nascent proteins labeled with DBCO = white, F-actin = magenta, DAPI = blue, scale bar = 50 μm). (**b**) Fluorescence of nascent protein staining within the cell body compared to the IgG-treated group (each dot = 1 cell as obtained from *n* = 3 biological replicates). (**c**) sGAG (left), DNA content (middle), and sGAG normalized by DNA content (right) in integrin α3-blocked cells compared to IgG, relative value = 100* (integrin-blocked/IgG-treated), each dot = 1 sample from *n* = 3 biological replicates. (**d**) Relative gene expression of phenotypic markers in the α3-treated group compared to the housekeeping *18S* and *GAPDH* (1^st^ Δ) and cells incubated with IgG control antibody (2^nd^ Δ), each dot = 1 biological replicate. (**e**) Heatmap showing percentage of cells with downregulated (50 % lower than IgG-treated cells) or upregulated (50 % higher than IgG-treated cells) protein expression. For all data presented: error bars = standard deviation; **** *p* < 0.0001, * *p* < 0.05 based on results of unpaired one-tailed *t-*tests (comparing IgG-treated to integrin-blocked).

Using FUNCAT techniques, newly synthesized proteins were fluorescently labeled in cells treated with the IgG control or antibodies to target integrin α3 (Fig. 5a). Intracellular fluorescence, a surrogate for nascent protein production, was reduced when integrin α3 was blocked (*p* < 0.0001, Fig. 5b). Additionally, the area containing newly produced extracellular proteins was lower on average in the integrin α3-blocked group (data not shown). DMMB and Picogreen assays were performed to specifically examine production of sGAG. While DNA content was not significantly different between the IgG and α3 integrin groups (*p* = 0.46, Fig. 5c), total sGAG production and normalized sGAG production were reduced in the integrin-blocked samples (*p* = 0.047 and *p* = 0.025, respectively, Fig. 5c).

### Inhibition of integrin α3 alters NP cell phenotype

Blocking integrin α3 elicited sustained effects on cell phenotype at the gene level. The gene expression of all phenotypic markers was decreased compared to the isotype control (Fig. 5d; *ACAN*: *p* = 0.023; *COL2A1*: *p* = 0.025; *CDH2*: *p* = 0.036; and *GLUT1*: *p* = 0.019). Protein expression for several NP markers was also altered by blocking integrin α3, though heterogeneity was seen across the cell populations and thus the percentage of cells with upregulated (at least 50 % greater) and downregulated (at least 50 % lower) expression was quantified. *BASP1* expression was downregulated in 6 % of cells and cell clusters and upregulated in 11 % of cells and cell clusters, while N-cadherin was downregulated in 16 % of cells/clusters and upregulated in 5 % (Fig. 5e). In contrast, expression of cytokeratins (pan-KRT) was not differentially expressed following integrin blocking (Fig. 5e).

**Fig. 6.**
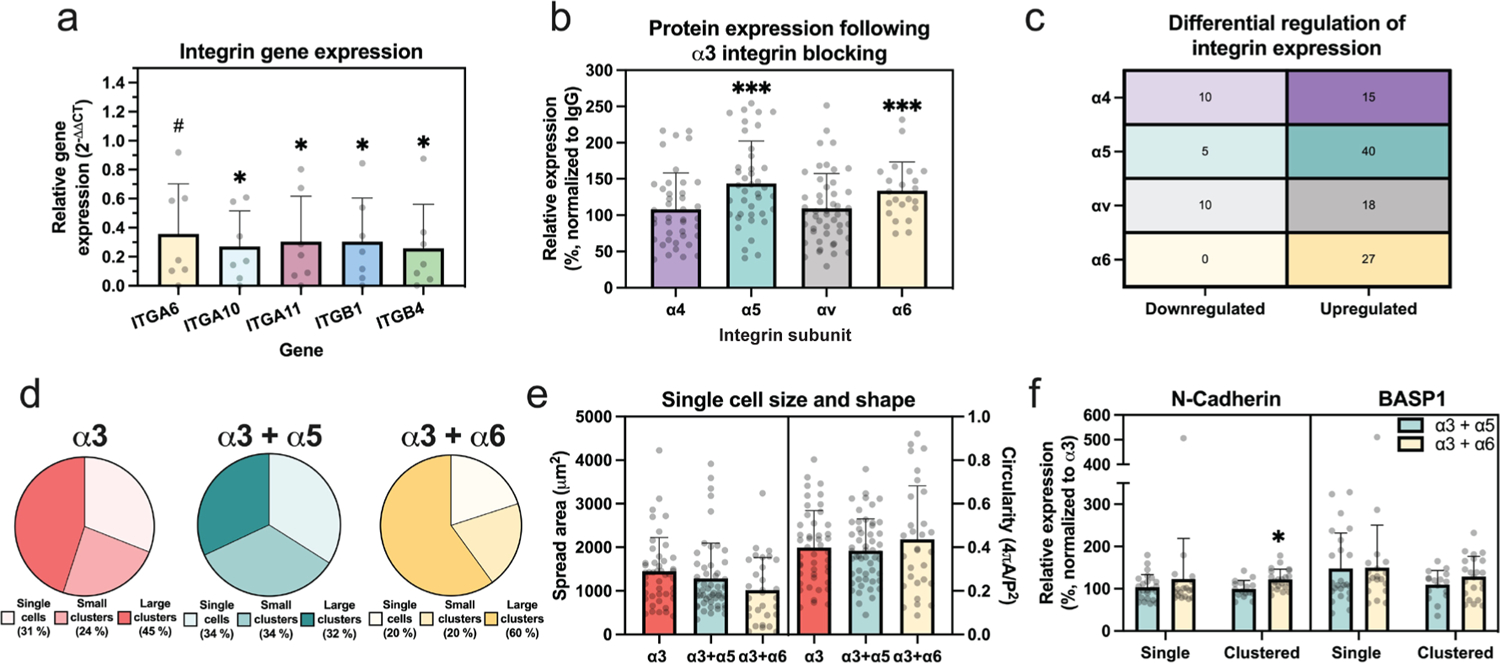
Blocking integrin α3 alters integrin expression. (**a**) Relative gene expression of integrin subunits in the α3-treated group compared to the housekeeping genes *18S* and *GAPDH* (1^st^ Δ) and cells incubated with IgG control antibody (2^nd^ Δ), each dot = 1 biological replicate. (**b**) Relative protein expression of integrin subunits in the α3-treated group compared to cells treated with IgG antibodies, relative expression = 100 × (integrin-blocked/IgG-treated), each dot = 1 cell from *n* = 3 biological replicates. (**c**) Proportion (%) of adherent cells subjected to integrin α3 blocking with upregulated (50 % higher than IgG-treated) or downregulated (50 % lower than IgG-treated) integrin subunits. (**d**) % of cells attaching to PEGLM as single cells, small clusters (2–3 cells), or large clusters (4+ cells) following inhibition of integrin α3 only or both integrins α3 + α5 or α3 + α6 (**e**) Spread area and circularity of single cells treated with integrin α3 only or both integrins α3 + α5 or α3 + α6, each dot = 1 cell from *n* = 3 biological replicates. (**f**) Relative protein expression of cells with both integrins α3 + α5 or α3 + α6 inhibited compared to cells with only α3 inhibited, relative expression = 100 × (dual integrin-blocked/α3 integrin-blocked), each dot = 1 cell from *n* = 3 biological replicates. For all data presented: error bars = standard deviation; *** *p* < 0.001, * *p* < 0.05, **#** *p* < 0.09 based on results of (**a**) paired two-tailed *t*-test of ΔCt values of integrin-blocked to IgG-treated (**b**) unpaired two-tailed *t*-test of MFI of integrin-blocked compared to IgG-treated (**e**,**f**) Ordinary one-way ANOVA with Dunnett’s *post hoc* test comparing dual integrin-blocked to single integrin-blocked (α3 only).

In addition to phenotypic markers, integrin expression was quantified following inhibition of integrin α3. Gene expression of integrin subunits was altered (*ITGA6 p* = 0.054; *ITGA10 p* = 0.036; *ITGA11 p* = 0.039; *ITGB1 p* = 0.037; *ITGB4 p* = 0.024; Fig. 6a) in α3-treated compared to IgG-treated cells. Compared to cells in the control group, blocking integrin α3 promoted the increased protein expression of integrin α5 (*p* = 0.0001) and α6 (*p* = 0.0009), but not α4 (*p* = 0.48) or αv (*p* = 0.30, Fig. 6b). Additional quantification revealed that 15–40 % of cells/cell clusters upregulated (50 % increase in protein expression) integrins α4, α5, αv, or α6 at the protein level following incubation with integrin α3-blocking antibodies (Fig. 6c), while downregulation (50 % lower protein expression) was seen in 0–10 % of the cells. As integrins α5 and α6 demonstrated increased expression following blocking of integrin α3, these two proteins were explored further to determine if they function to compensate for the loss of integrin α3.

When integrins α5 or α6 were blocked following 24 h of integrin α3 inhibition, altered distributions in cell morphology (single cells, small clusters, large clusters) were observed compared to cells where α3 alone was blocked (*p* < 0.00001, and *p* = 0.045, respectively, Fig. 6d). Increased small or large cell clusters were observed to form in cells with α3 and α5 or α3 and α6 blocked, respectively, compared to cells treated with the α3 antibody alone (Fig. 6d). In contrast, neither cell spread area nor circularity of single cells (Fig. 6e) were altered for either of the double blocked conditions compared to cells with α3 blocked alone (spread area: α3 + α5, *p* = 0.82; α3 + α6, *p* = 0.12; circularity: α3 + α5, *p* = 0.88; α3 + α6, *p* = 0.59). To explore whether cell-cell interactions are able to compensate for the additional loss of integrin function, protein expression was quantified for single cells and cell clusters subjected to the integrin blocking. N-cadherin expression was similar in single cells when inhibiting integrin α3 compared to cells with either inhibition of integrins α3 and α5 or α3 and α6 (*p* = 0.96 and *p* = 0.24 respectively; Fig. 6f left). In multi-cell clusters, N-cadherin expression was elevated when inhibiting integrins α3 and α6 (*p* = 0.045) but not when integrins α3 and α5 were inhibited (*p* = 0.9621; Fig. 6f left). Average expression of *BASP1* was not different between conditions where integrin α3 alone was blocked compared to blocking α3 and α5 or α3 and α6 for either single cells or cells in multi-cell clusters (Fig. 6f right; *p* > 0.26 for all comparisons).

## Discussion

Data from the present study demonstrated that integrin α3 plays a critical role in transducing cues from full-length laminin to adult human degenerative NP cells. When this subunit was inhibited, cell signaling and attachment were altered and the expression of markers of the healthy NP cell phenotype, that have previously been shown to be induced by the presence of laminin, were reduced. These findings illustrate the mechanisms by which previously developed laminin-presenting substrates have promoted cells to assume behaviors consistent with the healthy NP cell phenotype and can also be used to inform strategies for designing materials for treating intervertebral disc degeneration.

Laminins are found in the ECM of the developing and juvenile NP and NP cells have been shown to express integrins which interact with these proteins (Bridgen *et al*., 2013; Chen *et al*., 2009; Gilchrist *et al*., 2011b; Hayes *et al*., 2001; Nettles *et al*., 2004; Sun *et al*., 2017). Data from the present study corroborate prior findings that adult degenerative NP cells express integrins, specifically α3, α4, α5, αv, α6, and β1 subunits. While multiple integrin subunits are present at the membranes of the degenerative NP cells, results of the cell attachment assay indicate that blocking integrins α3 or α5 alone is sufficient to reduce initial cell attachment to PEGLM. Additionally, impairment of integrin β1 and, therefore, the heterodimers which form with integrin β1 (including α3β1, α6β1, α1β1, α2β1, α5β1, α4β1, and α10β1) significantly reduced NP cell attachment (Humphries *et al*., 2006; Hynes and Naba, 2012). These findings expand on previous literature which has demonstrated a role for multiple integrin subunits in mediating attachment to full-length laminins adsorbed to surfaces, and further confirms that adhesive sites within the laminin remain accessible to cells when the protein is conjugated to the biomaterial (Bridgen *et al.*, 2013; Francisco *et al.*, 2013).

Integrins not only promote attachment to ECM proteins, but also serve critical functions as mechanotransducers due to their intracellular interactions with the cytoskeleton and with hundreds of signaling molecules that regulate diverse cellular functions (Giancotti and Ruoslahti, 1999; Winograd-Katz *et al*., 2014; Yamada and Miyamoto, 1995). Studies in other cell types have demonstrated that modulating integrin function impacts cell signaling profiles (Chen *et al.*, 2016; Ferletta *et al.*, 2003; Velpula *et al.*, 2012). Quantification of protein phosphorylation in the present study similarly confirmed that engagement of degenerative NP cells with PEGLM initiates ERK 1/2 and GSK3β signal transduction pathways in an integrin-dependent mechanism. The data demonstrated that blocking integrin α3 reduced phosphorylation for both signaling molecules, whereas inhibiting the other integrin subunits tested either elicited less pronounced results or showed responses that differed between the two signaling pathways. Taking the outcomes of cell attachment and signaling together, it can be seen that integrin α3 is the primary mediator of adult degenerative NP cell interactions with PEGLM at early time points (≤ 2 h), events that are known to drive other cellular behaviors such as cell shape, biosynthesis, and phenotype.

At prolonged time points, cells incubated with integrin α3 blocking antibodies demonstrated alterations in the proportion of cells attaching to PEGLM as single cells or clusters and the single cells also showed increased cell spread areas and decreased circularity. Modulation of cell morphology has been previously shown to control phenotype and global transcriptome (Kilian *et al.*, 2010; McWhorter *et al.*, 2013). This effect has been confirmed in NP cells as well, with decreased intracellular tension and a concomitant rounding of cells on PEGLM being associated with increased expression of markers of the NP juvenile phenotype (Barcellona *et al*., 2020; Fearing *et al*., 2020; Francisco *et al*., 2014). In the present study, the altered cell shape observed in the α3-blocked cells may be due in part to the reduced ability of these cells to form elongated paxillin-rich focal adhesions. Paxillin is a member of the focal adhesion proteome, and is essential for the formation, disassembly, and maturation of FAs following integrin activation, allowing for the regulation of cytoskeletal organization, generation of contractile forces, and cell motility (Deakin and Turner, 2008; Larsen *et al.*, 2006; Miranti and Brugge, 2002; Yamada and Miyamoto, 1995). While the precise role of FA number and shape as it pertains to regulating NP cell phenotype is not well known, studies in other cell types have demonstrated a relationship between focal adhesion size/shape and cellular fate (Besser and Safran, 2006; Kim and Wirtz, 2013).

**Fig. 7.**
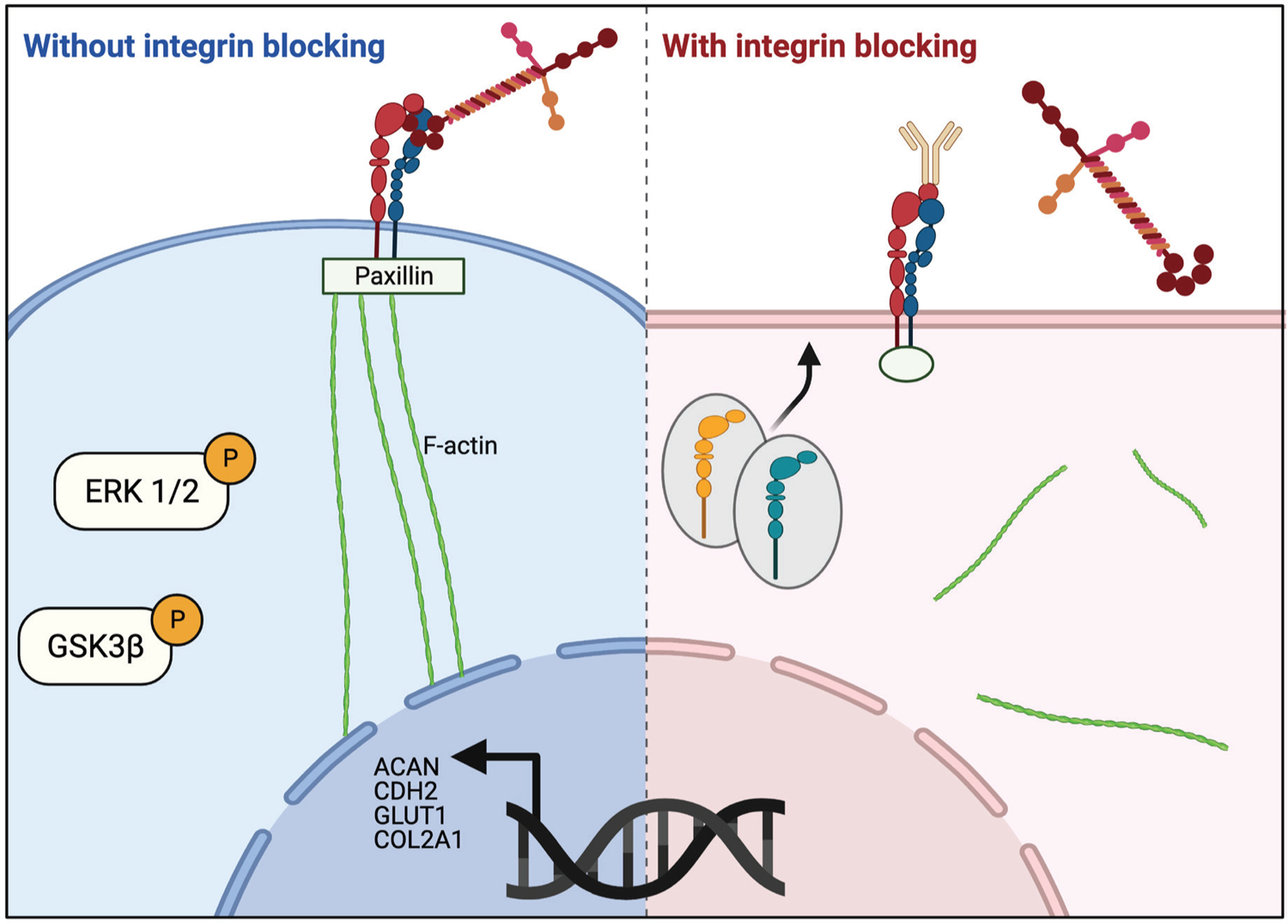
Proposed model describing the effects of integrin α3 inhibition on NP cell behaviors. (Left) When integrin α3 is able to bind to the laminin proteins in PEGLM, larger, elongated paxillin-positive focal adhesions are able to form which stabilize the connection between the extracellular domain and the cytoskeleton which demonstrates more aligned F-actin fibers and promotes increased cell circularity. Integrin engagement also promotes phosphorylation of ERK 1/2 and GSK3β and the transcription of phenotypic markers such as *ACAN*, *CDH2*, *GLUT1*, and *COL2A1*. (Right) In contrast, when cells are treated with antibodies which block integrin α3, cells are unable to bind efficiently to the laminin in PEGLM. More circular paxillin-positive focal adhesions form and the cytoskeleton exhibits reduced F-actin alignment and a larger, less round cell shape. Reduced integrin engagement decreases ERK 1/2 and GSK3β phosphorylation and phenotypic markers are transcribed less frequently. Additionally, integrin trafficking promotes increased expression of integrin α5 and α6. Image created using BioRender.com.

Integrin α3-mediated interactions with PEGLM controlled focal adhesion structures and actin alignment, which in turn modulated cell shape and phenotype (Fig. 7). Specifically, the data reveal that integrin α3 function is required for NP cells to re-express ACAN, CDH2, GLUT1, and COL2A1 and production of sGAGs, as has been previously shown for NP cells on hydrogels made from PEGLM. However, inhibition of integrin α3 was unable to eliminate expression of all the phenotype markers tested at the protein level. This finding may be in part due to the half-life of these proteins (Jaitovich *et al.*, 2008), but also suggests that other integrins (namely α5 and α6) may be trafficked to the membrane in order to compensate for the impairment of integrin α3 and may promote the continued protein expression of these markers (De Franceschi *et al.*, 2015; Kurakawa *et al.*, 2015; Mana *et al.*, 2020). The observation that dual inhibition of integrin subunits did not further alter cell spread area, circularity or protein expression in single cells provides additional support that integrin α3 may be the primary mediator for cell-ECM interactions for adult NP cells on PEGLM. However, as when integrin α3 alone was blocked, simultaneous inhibition of multiple integrin subunits (α3 and α5 or α3 and α6) further promoted the formation of cell-cell interactions. Concurrent inhibition of integrins α3 and α5 caused increased formation of small cell-clusters (where cell-ECM and cell-matrix interactions are relatively balanced); however, simultaneous inhibition of integrins α3 and α6 caused an increase in the presence of large cell clusters and thus, cell-cell interactions. Clustered cells with both integrins α3 and α6 blocked did not demonstrate differential BASP1 expression, but did show increased expression of N-cadherin, a protein known to facilitate increased cell-cell interactions (Hwang *et al.*, 2015; Marie, 2002). These findings are consistent with previous literature which has demonstrated that both cell-ECM and cell-cell adhesions can modulate cell behaviors during development and pathology, but that shifting the balance can modulate cell fate (Cosgrove *et al*., 2016; Crowder *et al*., 2016; Gilchrist *et al*., 2011a; Hwang *et al*., 2014; Lauffenburger and Griffith, 2001; Linde-Medina and Marcucio, 2018; McCain *et al.*, 2012; Ryan *et al.*, 2001). Additional studies, however, are required to further explore compensatory integrin-mediated mechanisms and to determine the relative roles of cell-cell and cell-matrix interactions in controlling NP cell behaviors. Data from the present study demonstrate that reduced cellular interactions with PEGLM (as caused by integrin inhibition) reduce this biomaterial’s ability to regulate the phenotype of adult degenerative NP cells in a mechanism that is not fully counteracted by increased formation of cell-cell interactions (Gilchrist *et al*., 2011a; Kurakawa *et al*., 2015; Nelson and Chen, 2003).

Previous findings utilizing PEGLM or laminin-mimetic peptides have demonstrated that NP cells can sense and respond to numerous features of the microenvironment including ligand presentation (Bridgen *et al*., 2017; Francisco *et al*., 2014; Gilchrist *et al*., 2011a), ligand density (Barcellona *et al*., 2020; Barcellona *et al.*, 2021), and bulk substrate stiffness (Gilchrist *et al*., 2011a), and that all of these parameters have roles in biomaterial design and tissue engineering approaches. In the present study, PEGLM-coated polystyrene was utilized in order to evaluate the impact of laminin presentation on NP cell phenotype, without introducing the variable of substrate stiffness, which can itself promote altered cell phenotypes. Similarly, this study presents the results of 2D culture and thus future studies will be needed to confirm the roles of integrin subunits during 3D culture and in the *in vivo*, mechanically loaded, native environment. Another limitation of the present study is the use of only degenerative NP cells, and thus the inability to determine differences in mechanotransduction between degenerative and healthy NP cells. Previous studies have demonstrated that integrin expression may be altered by pathology and severity of degeneration; however, additional research is needed to determine if and how integrin expression varies and the contributions of integrins to the degenerative cascade in the disc (Kanda *et al*., 2020; Kurakawa *et al*., 2015; Le Maitre *et al*., 2009; Xia and Zhu, 2008). Furthermore, studies which overexpress or exogenously activate integrin α3 subunits may provide insight into additional therapeutic options for preventing or treating disc disease.

## Conclusion

The data presented demonstrate that in the absence of integrin α3-mediated engagement with laminin, the cytoskeletal organization, gene expression of phenotypic markers, and biosynthesis of adult degenerative NP cells were significantly altered. Furthermore, blocking other integrin subunits in addition to α3 resulted in changes to cell morphology but overall phenotype was unaltered by continued integrin inhibition. This suggests that the α3 subunit plays a critical role in mediating phenotype for adult degenerative NP cells in culture on PEGLM. Together, these data demonstrate a mechanism of action for PEGLM, a clinically relevant material which can be used in the treatment of degenerative NP.

## Figures and Tables

**Table 1.** Antibodies used for integrin blocking experiments and corresponding isotype control. MilliporeSigma and Sigma-Aldrich, St. Louis, MO, USA; Thermo Fisher Scientific and Fisher Scientific, Waltham, MA, USA; Invitrogen, Carlsbad, CA, USA; BioLegend; San Diego, CA, USA; SouthernBiotech; Birmingham, AL, USA.

Integrin subunit	Anti-integrin antibody (20 μg/mL)	Control IgG antibody (20 μg/mL)
α3	Clone P1B5, azide free product: MAB1952Z (MilliporeSigma)	Mouse IgG1, azide free product: MA110407 (Thermo Fisher Scientific)
α4	Clone 9F10, azide free product: 50-143-40 (Fisher Scientific)	Mouse IgG1, azide free product: MA110407 (Thermo Fisher Scientific)
α5	Clone P1D6, azide free product: MAB1956Z (Sigma-Aldrich)	Mouse IgG3, azide free product: 0105-01 (SouthernBiotech)
αv	Clone 272-17E6, azide free product: MABT207 (Sigma-Aldrich)	Mouse IgG1, azide free product: MA110407 (Thermo Fisher Scientific)
α6	Clone GoH3, azide free product: MAB16884, (Thermo Fisher Scientific), 313637 (BioLegend)	Rat IgG2a, azide free product: 400516 (BioLegend), PIPA533214 (Fisher Scientific)
β1	Clone AIIB2, azide free product: MABT409 (MilliporeSigma)	Rat IgG1, azide free product: 400414 (BioLegend)

**Table 2.** Antibodies used to detect markers of NP cell phenotype.

Target	Species; dilution	Manufacturer
N-cadherin	Mouse; 1 : 100	Sigma-Aldrich
BASP1	Rabbit; 1 : 100	Abcam
Pan Cytokeratin	Mouse; 1 : 100	Sigma-Aldrich
Integrin *α*3	Rabbit; 1 : 100	Sigma-Aldrich

## List of Abbreviations

ACAN: aggrecan
ANOVA: analysis of variance
BASP1: brain acid soluble protein 1
BSA: bovine serum albumin
COL2A1: collagen type II alpha 1 chain
CDH2: N-cadherin
DAPI: 4’-6-diamidino-2-phenylindole
DBCO: dibenzylcyclooctyne
DMEM: Dulbecco’s modified Eagle’s medium
DMMB: dimethylmethylene blue
ECM: extracellular matrix
EDTA: ethylenediamine tetraacetic acid
ERK: extracellular-signal-regulated kinase
FA: focal adhesions
FBS: fetal bovine serum
FUNCAT: functional noncanonical amino acid tagging
GAPDH ?: glyceraldehyde 3-phosphate dehydrogenase
GLUT1: glucose transporter 1
GSK: glycogen synthase kinase
HEPES: 4-(2-hydroxyethyl)-1-piperazineethanesulfonic acid
IgG: immunoglobulin G
IVD: intervertebral disc
LM: laminin
MAPK: mitogen-activated protein kinase
MFI: mean fluorescence intensities
MR: magnetic resonance
NP: nucleus pulposus
PBS: phosphate-buffered saline
PEG: (poly)ethylene glycol
PEGLM: laminin-111-functionalized polyethylene glycol
PFA: paraformaldehyde
qPCR: quantitative polymerase chain reaction
RLT: buffer a lysis buffer for lysing cells and tissues
RT-qPCR: quantitative reverse transcription PCR
sGAG: sulfated glycosaminoglycan
YAP: Transcriptional coactivator YAP1
